# Past, Present, and Future of Viral Vector Vaccine Platforms: A Comprehensive Review

**DOI:** 10.3390/vaccines13050524

**Published:** 2025-05-15

**Authors:** Justin Tang, Md Al Amin, Jian L. Campian

**Affiliations:** 1 Department of Biomedical Science, University of Guelph, Guelph, ON N1G 2W1, Canada; 2 Department of Oncology, Mayo Clinic, Rochester, MN 55905, USA; amin.mdal@mayo.edu (M.A.A.); campian.jian@mayo.edu (J.L.C.)

**Keywords:** viral vector vaccines, adenovirus vectors, Modified Vaccinia Ankara (MVA), vesicular stomatitis virus (VSV), adeno-associated virus (AAV), immunogenicity, pre-existing immunity, prime-boost strategies, synthetic biology, vector engineering, manufacturing scale-up, regulatory challenges, vaccine safety, COVID-19, pandemic preparedness

## Abstract

Over the past several decades, viral vector-based vaccines have emerged as some of the most versatile and potent platforms in modern vaccinology. Their capacity to deliver genetic material encoding target antigens directly into host cells enables strong cellular and humoral immune responses, often superior to what traditional inactivated or subunit vaccines can achieve. This has accelerated their application to a wide array of pathogens and disease targets, from well-established threats like HIV and malaria to emerging infections such as Ebola, Zika, and SARS-CoV-2. The COVID-19 pandemic further highlighted the agility of viral vector platforms, with several adenovirus-based vaccines quickly authorized and deployed on a global scale. Despite these advances, significant challenges remain. One major hurdle is pre-existing immunity against commonly used vector backbones, which can blunt vaccine immunogenicity. Rare but serious adverse events, including vector-associated inflammatory responses and conditions like vaccine-induced immune thrombotic thrombocytopenia (VITT), have raised important safety considerations. Additionally, scaling up manufacturing, ensuring consistency in large-scale production, meeting rigorous regulatory standards, and maintaining equitable global access to these vaccines present profound logistical and ethical dilemmas. In response to these challenges, the field is evolving rapidly. Sophisticated engineering strategies, such as integrase-defective lentiviral vectors, insect-specific flaviviruses, chimeric capsids to evade neutralizing antibodies, and plug-and-play self-amplifying RNA approaches, seek to bolster safety, enhance immunogenicity, circumvent pre-existing immunity, and streamline production. Lessons learned from the COVID-19 pandemic and prior outbreaks are guiding the development of platform-based approaches designed for rapid deployment during future public health emergencies. This review provides an exhaustive, in-depth examination of the historical evolution, immunobiological principles, current platforms, manufacturing complexities, regulatory frameworks, known safety issues, and future directions for viral vector-based vaccines.

## 1. Introduction

Vaccines represent one of the greatest triumphs in public health, having successfully eradicated smallpox and dramatically reduced the incidence of poliomyelitis, measles, and other infectious diseases. Traditional vaccine approaches, such as inactivated or live-attenuated vaccines, have proven immensely effective against many pathogens. However, certain infections like HIV/AIDS, malaria, and tuberculosis, continue to pose formidable challenges to conventional vaccine designs [1]. Moreover, newly emerging zoonotic pathogens, exemplified by the Ebola virus, Zika virus, and coronaviruses (e.g., SARS-CoV-2) serve as a reminder that continuous innovation in vaccine technology is essential.

Along with this, viral vectors, genetically engineered viruses that can deliver foreign genes into host cells, have emerged as a powerful strategy for prophylactic and therapeutic vaccine development [2]. The concept is relatively straightforward but has profound immunological implications. By harnessing the biology of viruses, which naturally infect cells and induce robust immune responses, researchers can engineer harmless or attenuated viral backbones to express antigens from target pathogens. This approach often yields potent T-cell and B-cell responses that can exceed those elicited by protein-based or inactivated vaccines [3].

Since the late 20th century, scientists have explored a spectrum of viral backbones, from poxviruses like vaccinia and Modified Vaccinia Ankara (MVA), to adenoviruses, retroviruses, alphaviruses, and rhabdoviruses. Their applications extend not only to infectious diseases but also to cancer immunotherapy and beyond. Most notably, the COVID-19 pandemic underscored the impact of viral vector platforms on global health. Adenovirus-based COVID-19 vaccines, such as ChAdOx1 nCoV-19 and Ad26.COV2.S, were developed, tested, and authorized at unprecedented speed, demonstrating the adaptability of this platform in crisis scenarios [4].

Yet this success must be balanced against a host of unresolved challenges, including pre-existing immunity against the viral vector backbone, rare adverse events such as vaccine-induced immune thrombotic thrombocytopenia (VITT), manufacturing scale-up constraints, distribution issues, and the broader social-ethical complexities of ensuring equitable access to novel vaccines [5]. In parallel, breakthroughs in synthetic biology, computational antigen design, and advanced bioengineering promise next-generation viral vectors that are safer, more immunogenic, and more easily deployable.

Despite the large number of excellent papers that have appeared since 2020, the existing literature still shows three major blind spots. **First, most recent reviews are “platform-centric” (e.g., adenovirus-only, poxvirus-only) and therefore do not compare safety, immunogenicity, manufacturing, and regulatory hurdles across vector families in a single analytical framework. *Second, discussions of viral-vector scale-up, supply-chain fragility, and equitable distribution are usually treated in grey-literature policy briefs rather than in peer-reviewed scientific reviews, leaving a knowledge gap at the interface of bench science and global implementation. Third, only a handful of papers interrogate how post-COVID pharmacovigilance data (e.g., vaccine-induced immune thrombotic thrombocytopenia) should reshape vector design principles going forward.

The goal of this review is to provide a comprehensive, deeply detailed perspective on the current state and future directions of viral vector-based vaccines. We begin by tracing the historical evolution of viral vectors, then delve into the immunological fundamentals, the characteristics of different platforms, manufacturing intricacies, safety, and regulatory considerations, and the prospects for next-generation strategies. Our review makes four specific contributions: 1. it juxtaposes head-to-head data for the eight most widely used vector backbones, enabling a direct comparison that is missing from prior platform-specific reports; 2. it integrates manufacturing economics and regulatory pathways into the scientific narrative, thereby linking laboratory feasibility to real-world deployability; 3. it synthesizes the first full tranche of post-licensure safety signals—including VITT and anti-vector immunity—from more than one billion administered doses; and 4. it proposes a forward-looking research agenda that couples synthetic-biology advances with lessons learned on global access and public trust. By filling these gaps, the present review aims to serve as a one-stop, “bench-to-field” resource for researchers, developers, regulators, and implementation scientists working on next-generation viral-vector vaccines.

## 2. Historical Perspectives and Evolution of Viral Vector Vaccines

The foundational idea that viruses could be harnessed as carriers of heterologous antigens was already taking shape by the 1980s. Early experiments demonstrated that recombinant poxviruses, particularly vaccinia, could protect animal models against heterologous pathogens, thus providing a critical proof-of-concept for vector-based vaccines [6]. Researchers recognized the unique capacity of poxviruses to harbor large transgenes without compromising their replication or immunogenicity. This approach paved the way for a new field where the specificity of the immune response could be engineered rather than passively accepted. A pivotal moment in the historical development of viral vectors was the eradication of smallpox through widespread vaccination with the vaccinia virus. The success of this campaign, which ultimately resulted in the global elimination of a devastating disease, highlighted the safety and immunogenic capacity of vaccinia-based immunization in large populations [7]. Following smallpox eradication, researchers turned to modified derivatives like Modified Vaccinia Ankara (MVA), which offered a safer and more attenuated backbone while retaining the strong immunogenic qualities of vaccinia.

From the mid-to-late 1990s into the early 2000s, adenovirus-based vectors gained traction. Early clinical investigations, particularly those targeting HIV, showcased the advantages of adenovirus: easy manipulation, high-level gene expression, and potent T-cell responses [8]. However, the STEP trial for an HIV vaccine using Ad5 encountered an obstacle that reverberates to this day: pre-existing immunity in humans significantly dampened vaccine efficacy [8]. This finding catalyzed efforts to seek alternative serotypes (e.g., Ad26, Ad35) or even nonhuman adenoviruses (e.g., chimpanzee adenoviruses, such as ChAdOx1) to circumvent widespread immunity. Beyond poxviruses and adenoviruses, a growing body of work in the late 1990s and early 2000s explored vectors derived from vesicular stomatitis virus (VSV), alphaviruses, measles virus, lentiviruses, and more [9]. Each vector family offered unique immunological properties and manufacturing feasibility. For instance, alphaviruses provide strong self-amplifying RNAs capable of robust antigen expression; lentiviruses promise sustained antigen expression and potent T-cell induction, making them attractive for certain therapeutic vaccine concepts [10].

The licensing of rVSV-ZEBOV for Ebola virus disease prevention provided a landmark success, confirming that viral vectors could be safely deployed in outbreak settings and achieve high efficacy [9]. Subsequently, the COVID-19 pandemic saw adenovirus-based vaccines receive emergency use authorization worldwide, serving hundreds of millions of doses in record time [11,12]. These events collectively help show the agility and potential of viral vector technology in responding to both chronic and acute disease threats.

## 3. Classification and Characteristics of Common Viral Vector Platforms

Adenoviruses remain one of the most commonly used vectors due to their robust immunogenicity, ease of manufacturing, and ability to induce both T-cell and B-cell responses [13,14]. Adenoviruses are non-enveloped, double-stranded DNA viruses with a broad host cell tropism. Initial vaccine designs predominantly utilized human adenovirus serotype 5 (Ad5). However, high global seroprevalence of Ad5-specific neutralizing antibodies often compromises vaccine efficacy [15]. Clinical trials for HIV and other pathogens highlighted how pre-existing immunity could limit the effectiveness of Ad5-based vaccines. Consequently, alternative serotypes with lower pre-existing immunity rates, such as Ad26 (e.g., Ad26.COV2.S) and simian adenoviruses like ChAdOx1, have come to the forefront [16,17]. Despite their promise, adenovirus vectors have encountered rare but serious adverse events, specifically VITT in the context of COVID-19 vaccines. Nevertheless, adenovirus vectors remain a mainstay in the vaccine development landscape due to their scalability and track record across multiple clinical trials [18]. In addition, thermostability and established industrial processes for adenovirus production constitute significant advantages.

Adeno-associated viruses (AAVs) are small, non-enveloped viruses with single-stranded DNA genomes. They are renowned for their low pathogenicity and episomal persistence in non-dividing cells, traits that have made them a leading platform in gene therapy [19]. For vaccine applications, AAV’s advantages include relatively stable gene expression and a good safety profile. However, immunogenicity tends to favor humoral responses more than robust T-cell responses, and AAV’s limited cargo capacity (~4.5 kb) may be restrictive for certain antigen designs [20]. Another key issue for AAV-based vaccines is pre-existing immunity to naturally circulating AAV serotypes, which can neutralize the vector before it infects target cells [21]. The high prevalence of anti-AAV antibodies in the general human population could hamper efficacy or limit the applicability of certain serotypes. Researchers have begun exploring a range of less common AAV serotypes or engineered capsids to avoid neutralization, but widespread adoption in prophylactic vaccines for infectious diseases is still under development [22].

Poxvirus vectors have a long history in vaccinology, originating from the success of vaccinia virus in eradicating smallpox. The attenuated derivative MVA (Modified Vaccinia Ankara) is particularly noteworthy for its excellent safety profile: it replicates poorly in most human cells but retains a robust immunogenic capacity [23]. Poxviruses have large genomes, which offer the capacity to incorporate multiple or complex antigens. This makes them attractive for delivering multivalent vaccines or prime-boost strategies targeting multiple targets, such as HIV, malaria, and TB antigens [24]. Nevertheless, manufacturing poxviruses can be more complex than adenovirus or AAV, partly due to their large size and cytoplasmic replication cycle [25]. Despite these technical hurdles, the strong track record and extensive clinical data on MVA-based vectors have kept poxviruses central to the viral vector conversation.

VSV is an enveloped, negative-sense RNA virus that has garnered attention for its ability to induce potent cellular and humoral immunity. Its replication typically occurs in the cytoplasm, reducing the risk of insertional mutagenesis. A key advantage is the possibility of exchanging the VSV glycoprotein with that of other viruses, effectively pseudotyping the vector to display foreign antigens on the virion surface [26]. The rVSV-ZEBOV Ebola vaccine capitalized on this property, expressing the Ebola glycoprotein to induce targeted immunity while relying on VSV for viral replication and immunogenic cues [9]. However, VSV-based vaccines must strike a balance between adequate replication for strong immunogenicity and sufficient attenuation for safety. Concerns about potential neurovirulence or unintended tissue tropism persist, prompting ongoing research into further attenuation strategies [27].

The measles virus (MV) has been leveraged to create recombinant vaccines based on the well-established, highly attenuated measles vaccine strains. These vectors elicit strong, durable humoral and cellular immunity [28]. Notably, pre-existing immunity to measles in most vaccinated populations does not necessarily abrogate the vector’s ability to express a foreign antigen. In addition, measles vectors have shown promise in mucosal immunization, an important advantage for respiratory pathogens [29]. Other paramyxoviruses, such as Newcastle disease virus (NDV), are under exploration for vaccine development, particularly for zoonotic infections and even for certain forms of oncolytic therapy [30]. However, similar to other RNA viruses, ensuring genetic stability and controlling attenuation remain pressing challenges.

Alphavirus vectors, including those derived from Sindbis virus or Semliki Forest virus, often use self-replicating RNA replicons to achieve high levels of antigen expression [31]. The potent innate immune response triggered by alphavirus replicons can be a double-edged sword, leading to strong immunogenicity but occasionally excess reactogenicity. Research efforts focus on refining replication fidelity, and attenuation, and improving the ease of large-scale manufacturing. Self-amplifying RNA (saRNA) vaccines, which can be considered a sub-class of alphavirus replicon technology, have attracted attention due to their ability to deliver genetic material in a platform that can reduce dosage requirements while enhancing immunogenicity [32]. These approaches may offer a partial solution to the manufacturing complexities of large volumes of conventional viral particles.

Among the retroviruses, lentiviruses (most notably HIV-1-derived) stand out for their capacity to integrate into host genomes, thereby supporting prolonged antigen expression [33]. While integration raises concerns about insertional mutagenesis, the development of integrase-defective lentiviral vectors (IDLVs) has circumvented many of these safety issues. IDLVs can remain in an episomal state, offering persistent antigen expression without the genetic risk associated with full integration [34]. However, lentiviral vector (LV) vaccine development faces practical hurdles, including complex manufacturing protocols, higher production costs, and the need for stringent biosafety level (BSL) containment [35]. Despite these challenges, lentiviral vectors remain attractive for specialized applications like therapeutic cancer vaccines, where sustained antigen presentation to T cells may be critical for mounting an effective immune response.

**Figure 1:**
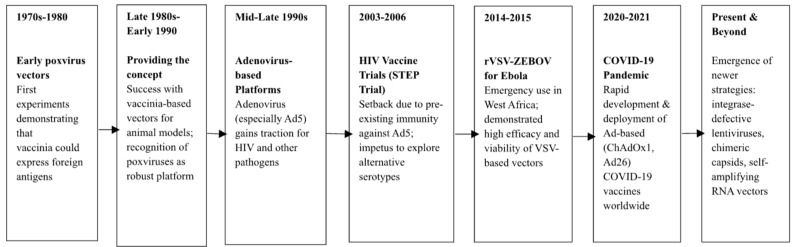
Key milestones in the development of viral vector vaccines. The timeline highlights the progression from early poxvirus experiments to modern platforms used during the COVID-19 pandemic.

Recent research has explored insect-specific flaviviruses (ISFs) and other arthropod-restricted viruses that naturally do not replicate in vertebrate cells. By genetically engineering these ISFs to carry structural proteins of human-pathogenic arboviruses (e.g., dengue, Zika, chikungunya), scientists aim to develop safe, replication-deficient vaccines for humans [36]. Early preclinical data are encouraging, demonstrating immunogenicity without risk of vector-induced disease [37]. Although still in the early stages, these platforms may offer a valuable alternative for controlling mosquito-borne infections. These events collectively illustrate the agility and potential of viral vector technology in responding to both chronic and acute disease threats (Figure 1, Table 1).

## 4. Mechanisms of Immunogenicity and Immune Response Induction

One of the main reasons viral vector vaccines can outperform certain traditional vaccines is their strong induction of innate immunity. Viral vector components, such as capsid proteins, envelope glycoproteins, or viral genetic material, are detected by pattern recognition receptors (PRRs) on or within host cells [38]. Toll-like receptors (TLRs) in endosomes may recognize viral nucleic acids, while RIG-I-like receptors (RLRs) in the cytoplasm sense RNA from replicating vectors [39]. The outcome is a cytokine cascade that includes type I interferons, crucial for priming the adaptive immune response.

Following transduction, the viral vector delivers its genetic payload into host cells, leading to intracellular antigen processing and presentation on MHC class I molecules [40]. This direct presentation pathway is especially potent at eliciting CD8+ cytotoxic T lymphocytes (CTLs). Simultaneously, antigens taken up by professional antigen-presenting cells (APCs) can be presented on MHC class II, promoting robust CD4+ T helper responses. Collectively, these pathways result in the activation of B cells for antibody production, along with cytotoxic T-cell-mediated clearance of infected or malignant cells.

Different viral vectors induce distinct profiles of innate and adaptive responses [41]. For instance, adenovirus vectors are known for rapidly triggering Type I interferon responses, which can be beneficial for immunogenicity but may also contribute to vector-directed inflammatory effects. Alphaviruses tend to produce strong innate responses through dsRNA intermediates, sometimes leading to robust reactogenicity. MVA, on the other hand, typically exhibits a favorable safety profile with considerable immunogenicity, making it appealing in prime-boost regimens.

The route of administration can greatly influence the immune response. While intramuscular (IM) injection primarily induces systemic IgG and T-cell responses, intranasal or oral delivery can help establish mucosal IgA and tissue-resident memory T cells at critical sites of pathogen entry [42]. For respiratory viruses like SARS-CoV-2 or influenza, mucosal immunity could be a key factor in blocking transmission. However, delivery through mucosal routes may require higher vector doses or formulations that protect the virus from neutralization in the mucosal environment [43].

**Figure 2:**
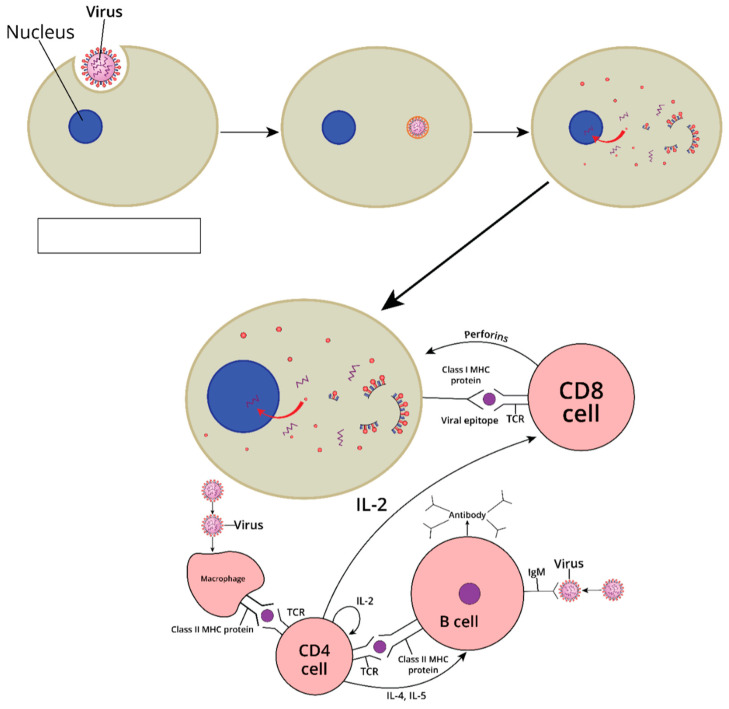
Schematic overview of immune response induction by viral vector vaccines. Viral vectors facilitate antigen delivery into host cells (top panel), leading to intracellular processing and presentation on MHC class I molecules, which activates cytotoxic CD8+ T cells (bottom right). Antigens taken up by antigen-presenting cells (APCs) like macrophages are presented on MHC class II molecules, activating CD4+ T helper cells. These helper cells provide signals (e.g., IL-2, IL-4, IL-5) that support B cell activation, proliferation, and differentiation into antibody-producing plasma cells (bottom left).

Heterologous prime-boost regimens, where an individual receives one type of viral vector (or vaccine platform) for the prime and another for the boost, can circumvent issues like anti-vector immunity and often lead to enhanced immunogenicity [44]. For example, an Ad prime followed by an MVA boost may yield higher T-cell frequencies than repeated dosing with the same vector. This strategy has been studied in HIV vaccine trials, as well as in the context of emerging pathogens [45]. It also became a relevant practice during the COVID-19 pandemic, where “mix-and-match” regimens proved beneficial for certain populations [46]. Collectively, these pathways result in the activation of B cells for antibody production, along with cytotoxic T-cell-mediated clearance of infected or malignant cells (Figure 2).

## 5. Current Trends in Preclinical and Clinical Development

Viral vector vaccines are frequently at the forefront of emergency responses to new outbreaks. For emerging pathogens like the Lassa virus, Nipah virus, and Marburg virus, the ability to quickly engineer vectors by swapping out antigenic genes has accelerated the path from pathogen discovery to clinical testing [47]. The success of rVSV-ZEBOV for Ebola illustrated how a single vector platform could be adapted to express different glycoproteins for various hemorrhagic fever viruses, simplifying the vaccine development pipeline [9]. In the case of coronaviruses, existing adenovirus vector backbones were rapidly repurposed during the COVID-19 crisis, illustrating how prior platform development laid the groundwork for quick pivoting to novel pathogens [48]. Looking ahead, many research consortia now maintain “sleeping” vector constructs that can be swiftly adapted once a new virus of concern emerges.

There are multiple challenges in developing a safe and effective HIV vaccine. HIV envelops glycans which play a critical role in evading the immune system by hiding epitopes. HIV seeds into immune-privileged anatomic reservoir sites which help them to protect against immunity [49]. Moreover, HIV has Vif and Vpu proteins which antagonize viral restriction factors [49]. There are several trials are ongoing on HIV vaccination. A recent phase 3 trial (NCT03964415) featured Ad26Mos4.HIV, Ad26 vectored vaccine trial showed that vaccine is not effective in preventing HIV infections compared to placebo [50]. A new strategy uses germline targeting eOD-GT8 which is a nano particle coated with an enveloped protein that facilitates the production of neutralizing antibody response [50]. Two first inhuman trials are underway evaluating the safety and immunogenicity of such eOD-GT8 in HIV-uninfected adults [51]. Recent Phase 1/2 studies, including HVTN 105 and the APPROACH trial, suggest that heterologous prime-boost regimens using Ad26 followed by MVA can generate more robust and broader T-cell immunity compared to Ad5-based approaches, potentially bypassing pre-existing Ad5 immunity [51].

Despite the complexity of HIV immunology, viral vectors remain core components of ongoing vaccine research [52]. Early disappointment in adenovirus-based candidates prompted a shift toward alternative vectors (Ad26, MVA) or prime-boost strategies. Researchers are also investigating mosaic antigens that combine multiple HIV clades to elicit broader T-cell coverage [53]. While a definitive HIV vaccine remains elusive, the adaptability and immunogenicity of viral vectors remain central to the effort. Malaria vaccine development also utilizes viral vectors, particularly adenovirus or poxvirus backbones, to deliver sporozoite or blood-stage antigens [54]. Achieving partial protection could significantly reduce disease burden, and coupling vector-based approaches with novel adjuvants or anti-parasitic drug regimens may boost efficacy. Beyond HIV and malaria, vector-based vaccines for chronic infections like hepatitis C are in preclinical or early clinical stages, focusing on T-cell-mediated clearance mechanisms [55].

The malaria parasite has different stages in its lifecycle. For example, while pre-erythrocytic vaccines target liver-stage parasites preventing entry into the erythrocytic stage, the blood-stage vaccines target asexual parasites to reduce disease [56]. A new RTS,S regimens are being examined in malaria naïve adults a fractioned third dose and delayed dosing showed significantly increased efficacy against control human malarial infection. PfSPZ vaccines showed efficacy in Malian adults who received presumptive antimalarial treatment but showed no efficacy in Kenyan infants without prior antimalarial treatment [56]. PfRh5 formulated in AS01 reduces parasite multiplication during CHMI, AMA1. A prime-boost regimen of ChAd63 and MVA expressing ME-TRAP has shown partial protection (up to 67% sterile protection) in controlled human malaria infection trials, indicating the promise of adenovirus–poxvirus combination strategies [57].

Most of the ebola virus disease outbreaks originated in Middle Africa until 2013 but, from 2013 to 2016 ebola caused the largest outbreaks which was responsible for 28,652 infections and 11,325 deaths. Ebola virus, a member of the filo virus family contains glycoprotein GP1,2 which is responsible for major immunogenicity and is the target protein in most vaccines [58] The rSV-ZEBOV is such type of vaccine achieved nearly 100% efficacy in a ring vaccination trial during the 2015–2016 trial in Guinea (0 cases in the immediate vaccination group vs. 23 in the delayed group), showcasing the power of rapidly deployed viral vector platforms in epidemic control [8].

It is a replication-competent, recombinant vesicular stomatitis virus (rVSV) a vectored vaccine that was approved by the FDA [59].

A persistent challenge in influenza vaccine development is the rapid antigenic drift of circulating strains. As a result, annual vaccination campaigns must regularly update strain composition. Researchers hope to develop “universal influenza vaccines” that target conserved epitopes, such as the hemagglutinin (HA) stem, thereby offering cross-protection against a wide range of subtypes [60]. Viral vectors, including adenovirus and MVA, have shown promise in displaying these conserved domains. Computational antigen design combined with structural biology has further refined this approach, aiming to generate more stable antigen constructs that elicit broadly neutralizing antibodies [61].

Beyond infectious diseases, the capacity of viral vectors to induce robust T-cell responses makes them attractive platforms for therapeutic cancer vaccines. Glioblastoma (GBM), the most aggressive primary brain tumor in adults, exemplifies the unmet clinical need in this area, with median survival stubbornly fixed at ≈15 months despite maximal surgery, radiotherapy, and temozolomide [62]. Clinical trials are underway examining MVA or adenovirus vectors that encode tumor-associated antigens, such as prostate-specific antigen (PSA) or oncoproteins associated with HPV-induced malignancies. The synergy with immune checkpoint inhibitors, like anti-PD-1 or anti-CTLA-4 antibodies, may enhance T-cell infiltration and reduce tumor immune evasion [63]. Moreover, the advent of personalized cancer vaccines involves sequencing a patient’s tumor to identify unique “neoantigens,” which can then be encoded in a viral vector. Early-phase trials suggest this individualized approach can generate potent anti-tumor responses. A particularly appealing strategy for GBM is because glioblastoma stem cells sustain tumor heterogeneity, drive therapy resistance, and repopulate recurrent lesions. Early-phase trials suggest this approach can generate potent anti-tumour responses [64,65].

## 6. Manufacturing, Scale-Up, and Quality Control

One of the most formidable barriers to viral vector vaccine deployment is the scaling-up of manufacturing processes to supply global need. While academic or small-scale facilities can produce research-grade vectors, the transition to Good Manufacturing Practice (GMP)-compliant processes at a commercial scale is complex. Concerns revolve around process consistency, product purity, and adequate yields. Cell lines such as HEK293, PER.C6, or Vero cells are often the backbone of viral vector production. These cells must be grown in large-scale bioreactors, often single-use or fixed-bed systems, to achieve the required volume [66]. Success depends on optimizing cell density, culture media, and infection parameters to maximize vector titers.

Downstream processing typically involves chromatography, tangential flow filtration (TFF), or ultrafiltration to remove cellular debris, host-cell DNA, and other impurities while concentrating the viral product. Each step must be validated for efficiency and reproducibility, and every batch must meet rigorous specifications for potency, sterility, and absence of replication-competent revertants [67]. The presence of aggregates or contaminants can reduce efficacy or provoke unwanted immune responses.

Thermostability is a persistent challenge for many viral vector platforms. Most preparations require refrigerated (2–8 °C) or even ultra-cold (−20 °C to −80 °C) storage conditions. Maintaining these conditions worldwide, especially in remote or resource-limited settings, is logistically demanding and costly. Research on lyophilization or novel excipients aims to improve stability at higher temperatures. For instance, adding cryoprotectants, stabilizing sugars, or using advanced drying techniques can preserve vector integrity. If successful, such strategies could dramatically improve global vaccine distribution, particularly in low- and middle-income countries [68,69].

Regulatory agencies like the U.S. FDA require extensive documentation on manufacturing processes, including validated assays for vector identity, potency, purity, and safety. The absence of replication-competent virus (for replication-deficient vectors) and genetic stability over multiple passages are particularly important. In emergency scenarios, such as a pandemic, agencies may issue Emergency Use Authorizations (EUAs) that allow provisional distribution while additional data are collected. International harmonization of regulatory standards remains a work in progress, yet it is critical for efficient multi-regional approvals [70,71].

## 7. Safety and Efficacy Considerations

The first question in evaluating a viral vector vaccine is often: “Is it safe?” For non-integrating vectors (e.g., adenovirus, poxvirus, VSV), concerns center on residual pathogenicity or the emergence of replication-competent revertants in immunocompromised recipients. In the case of lentiviruses, integration can theoretically lead to insertional mutagenesis, although integrase-defective lentiviral vectors (IDLVs) have largely mitigated this risk [72]. Developers use a variety of attenuation strategies to reduce pathogenicity, from deleting virulence genes to incorporating conditional replication circuits that function only in specialized production cells. Each approach must undergo rigorous preclinical testing to confirm genetic stability and minimal reversion to virulence [66].

Pre-existing immunity, particularly relevant for Ad5 and other common adenovirus serotypes, can significantly lower vaccine immunogenicity by neutralizing the vector before it delivers its payload. This challenge is circumvented in multiple ways:

- Using alternative serotypes (Ad26, Ad35, or simian adenoviruses) [73]
- Employing poxvirus, VSV, or other unrelated vectors
- Adopting heterologous prime-boost regimens
- Engineering chimeric capsids with novel antigenic surfaces [74]

Each strategy adds complexity to vaccine design and may introduce new immunological or manufacturing considerations.

The global rollout of adenovirus-based COVID-19 vaccines illuminated the rare but serious phenomenon of vaccine-induced immune thrombotic thrombocytopenia (VITT) (also referred to as TTS, thrombosis with thrombocytopenia syndrome) [75]. Although extremely infrequent, VITT involves autoantibody formation (often anti-PF4 antibodies) that activate platelets, culminating in life-threatening thrombotic events. The precise mechanism remains under investigation, but proposed explanations include viral DNA interacting with platelet factor 4, or vector impurities driving an aberrant immune reaction [76]. It is a rare adverse effect of adenoviral vector-based SARS-CoV-2 (COVID-19) vaccines specially ChAdOX1 nCoV-19 and Ad26.COV2.S.

The risk of developing such complication is 1 case per 26,500 to 127,300 after the first COVID-19 vaccine dose [77]. The overall risk remains very low, and the benefit-risk ratio for preventing severe COVID-19 is still overwhelmingly favorable. Nevertheless, these events have prompted ongoing refinements in vector design, production purity, and pharmacovigilance. Vaccinology must remain vigilant for new safety signals and continue to refine manufacturing processes to minimize any immunogenic impurities.

While many viral vector vaccines generate robust initial responses, the longevity of protective immunity can vary. Factors influencing durability include vector dose, antigen design, route of administration, and the presence of immune-regulatory mechanisms. Repeated boosting with the same vector can be hampered by anti-vector immunity, leading to the exploration of IDLVs or prime-boost strategies with distinct platforms [78].

Although most of the vaccines follow homologous prime boast regimens some studies showed the hetelogous boost regimens produce more immunogenic response than the homologous ones. For this reason, CDC issued a guidance that supports mix and match heterologous COVID-19 vaccine strategy [79].

In some chronic infections or tumor settings, continuous antigen expression could risk immune tolerance or exhaustion. Researchers are thus looking into combining viral vector vaccination with immune modulators, such as checkpoint inhibitors (e.g., anti-PD-1) or cytokine adjuvants, to sustain T-cell functionality over time [80].

## 8. Innovations and Engineering Strategies in Vector Design

The recent surge in synthetic biology has introduced advanced methods of engineering viral genomes to include conditional replication systems and “kill” switches. For instance, temperature-sensitive mutations can render the virus replication-competent only under specific lab conditions, or small-molecule-inducible promoters can control antigen expression. Such “circuit designs” reduce the risk of horizontal transmission or uncontrolled spread in the environment, thereby enhancing the biosafety of live or replicating vectors [81]. Another strategy to amplify vaccine efficacy is codon optimization, adjusting the codon usage of the transgene to match host cell preferences, leading to higher levels of antigen expression [82]. Additionally, embedding molecular adjuvants (e.g., GM-CSF or cytokine genes) within the vector can direct immune responses toward a more potent cellular or humoral profile. Some vaccine developers incorporate consensus or “mosaic” antigens that capture a broader range of epitopes for hypervariable viruses like HIV or influenza [83].

Pseudotyping, swapping surface glycoproteins or capsid components, can allow a vector to infect specific cell types more efficiently or evade pre-existing immunity. For example, VSV can be pseudotyped with the Ebola glycoprotein or other viral envelopes to direct the immune response toward particular antigens [26]. Similarly, lentiviruses can be pseudotyped with the VSV-G protein to broaden tropism. Such modifications can be crucial in ensuring the vector preferentially infects certain APCs, like dendritic cells, thereby enhancing immunogenicity. Combination regimens that utilize more than two platforms, e.g., a prime with an alphavirus replicon followed by a boost with a poxvirus vector, then a final boost with a protein subunit, are being explored to fine-tune both T-cell and B-cell compartments. The COVID-19 crisis offered real-world data on mixing different vaccines (mRNA and adenovirus vectors), showing both immunologic and logistical advantages. Future pandemic preparedness strategies are likely to incorporate this flexible approach to ensure broad coverage and robust immunological memory [84].

## 9. Lessons Learned from the COVID-19 Pandemic

One of the most striking takeaways from the pandemic is how pre-existing adenovirus platforms could be rapidly adapted to address a novel pathogen, SARS-CoV-2 [4]. Years of foundational research on adenovirus vectors for Ebola, MERS, and HIV underpinned the swift development of ChAdOx1 nCoV-19 and Ad26.COV2.S [16,17]. This success underscores the importance of continuous R&D investments in prototype pathogen pipelines and plug-and-play vector technologies [84]. Despite the impressive development speed, bottlenecks in raw materials (e.g., filters, growth media), limited fill-finish capacity, and distribution challenges highlighted persistent vulnerabilities in the global vaccine supply chain. Expanding manufacturing footprints, especially in low- and middle-income countries, and diversifying supply sources emerged as urgent priorities. Partnerships between pharmaceutical companies, governments, and international organizations like the WHO played a key role but also revealed inequities in vaccine availability [85].

The rapid identification of VITT cases exemplified how robust post-marketing surveillance systems (e.g., VAERS in the U.S., EudraVigilance in the EU) can detect rare adverse events. Transparent communication about these risks, coupled with clear guidance, is critical in maintaining public trust [75]. The pandemic also showcased the complexities of balancing public health needs with evolving scientific data, as regulators and health authorities had to adapt recommendations swiftly. Constraints in vaccine supply and emerging variants of concern pushed many countries to adopt heterologous prime-boost schedules. Studies indicated that mixing an adenovirus prime with an mRNA booster (or vice versa) was immunologically robust and sometimes superior to homologous regimens [46]. This flexibility may remain essential for future outbreak responses where vaccine supply or variant-specific efficacy must be optimized.

## 10. Socioeconomic and Global Access Considerations

Despite unprecedented scientific achievements, the pandemic spotlighted gross inequities in vaccine distribution. Wealthy nations secured billions of doses early on, while lower-income countries often waited months or years to access adequate supplies. Viral vector vaccines, while generally more stable than mRNA counterparts, still encountered cold-chain hurdles. Programs like COVAX aimed to mitigate disparities, but technology transfer and regional manufacturing are recognized as more sustainable solutions for future pandemics [86].

The rapid scale-up of viral vector vaccine production can be hampered by intellectual property (IP) restrictions around vector platforms, proprietary manufacturing processes, or specialized reagents. Voluntary or compulsory licensing agreements, patent pools, and open-science collaborations have been proposed to accelerate knowledge sharing. Some vaccine developers, such as those behind ChAdOx1, engaged in broad partnerships to expand global manufacturing. However, the tension between profit-driven models and public health needs remains a substantial barrier [87].

Viral vector vaccines, like others, face vaccine hesitancy fueled by misinformation, cultural beliefs, or mistrust of public health institutions [88]. Transparent engagement with communities, especially in resource-limited settings, is essential to dispel myths and ensure that the benefits of vaccination are equitably realized. Tailored communication, translated into local languages and addressing specific cultural concerns, can help increase acceptance of novel vaccine platforms (Table 2).

## 11. Future Perspectives: Next-Generation Viral Vector Platforms

Future innovations likely hinge on integrating synthetic biology with computational antigen design. This synergy allows for the construction of fully synthetic viral backbones with minimal extraneous genetic elements, reduced immunogenic footprints, and controllable replication dynamics [89]. Additionally, structural bioinformatics can pinpoint conserved epitopes within highly mutable pathogens like influenza, HIV, or coronaviruses, guiding the design of universal or broadly protective vaccines. The success of mRNA vaccines during COVID-19 has reignited interest in self-amplifying RNA vectors derived from alphaviruses. By coupling RNA-based technology with viral vector concepts, researchers hope to maximize immunogenicity while reducing dose requirements and simplifying manufacturing [90]. “Plug-and-play” approaches, where a standardized backbone is quickly loaded with a new antigen, are very important to pandemic preparedness strategies [91].

Advances in genomics and bioinformatics have catapulted the concept of personalized vaccines, particularly for cancer but potentially also for rapidly evolving pathogens [90]. Through sequencing-based approaches, unique patient-specific neoantigens (in oncology) or variant-specific immunodominant regions (in emerging pathogens) can be engineered into viral vectors. Preliminary clinical data suggest that such personalized constructs can drive potent immune responses with minimal off-target effects. Needle-free delivery approaches (e.g., intranasal sprays, oral capsules, or microneedle patches) offer potential advantages for certain viral vector vaccines [92]. Apart from improving user compliance (especially in pediatric or needle-phobic populations), these routes can induce mucosal immunity. If carefully validated, such strategies could revolutionize mass immunization campaigns by reducing logistical burdens, needlestick injuries, and medical waste.

## 12. Discussion and Outlook

Viral-vector vaccines have moved from “proof-of-principle” curiosities to indispensable instruments of both routine immunization and rapid outbreak response. Their greatest strength is conceptual simplicity married to extraordinary modularity: once a safe viral backbone is in hand, swapping in a new antigen cassette is largely a molecular-cloning exercise rather than an entirely new product cycle. That plug-and-play logic, refined over two decades, enabled adenovirus-based COVID-19 vaccines to reach billions of people within a single calendar year—an achievement that would have been inconceivable with classical egg-based or protein-subunit technologies. Yet the same experience laid bare the platform’s brittle points: supply chains faltered on single-use plastics and filtration membranes, regional manufacturing gaps delayed access, and rare adverse events such as vaccine-induced thrombotic thrombocytopenia demanded real-time pharmacovigilance and transparent risk communication. Thus, the story of viral-vector vaccinology is no longer only about immunology or virology; it is equally about logistics, global equity, and public trust.

At the scientific level, three converging trends are reshaping the next generation of vectors. First, advances in synthetic biology allow “genome-minimal” backbones that eliminate non-essential or potentially reactogenic genes, improving safety profiles while freeing genetic space for larger or multiple antigens. Second, deep learning–driven antigen design produces stabilized, conformationally correct immunogens that maintain potency even when delivered from episomal or attenuated vectors with limited expression windows. Third, intrinsic adjuvantation is being engineered directly into vectors—whether through codon-optimized cytokine payloads, co-expressed chemokines that recruit dendritic cells, or capsid modifications that engage favorable pattern-recognition receptors—to decouple immunogenicity from high dose requirements. Collectively, these innovations aim to generate “low-reactogenicity, high-immunogenicity” constructs that can be manufactured at lower cost and administered at lower volume, expanding the practical reach of mass campaigns.

Safety, historically framed as an exercise in proving an absence of integration or reversion to virulence, is evolving into a broader concept of “immunological homeostasis.” Developers must now anticipate how vector genomes, residual host-cell proteins, and formulation excipients interact with the innate immune system to create either balanced adjuvantation or pathological inflammatory cascades. In parallel, regulators are shifting from binary go/no-go paradigms toward continuous-learning models that integrate real-world evidence, genomic surveillance of viral variants, and post-marketing immune-profiling data. Agile regulatory science will be critical if vectors are to keep pace with hyper-mutable pathogens such as influenza, HIV, or coronaviruses without requiring a full re-licensure each season.

Manufacturing remains the decisive bottleneck for equitable impact. Platform processes in suspension cell lines, single-use bioreactors, and closed downstream skids have largely solved consistency and biosafety in high-income settings, but the scale still concentrates on a handful of facilities. The next leap will come from true regionalization—technology-transfer packages that embed process analytics, quality-by-design templates, and digital twin monitoring so that a facility in Nairobi or São Paulo can run the same validated workflow executed in Boston or Basel. Thermostabilized formulations and needle-free delivery devices will further uncouple vaccine access from cold-chain stringency and skilled injector availability, thereby broadening the reach of emergency ring-vaccination strategies to remote or insecure areas.

From a public health vantage, viral-vector platforms sit at the crossroads of two diverging societal forces: growing vaccine hesitancy and rising expectations for personalized medicine. Because vectors often evoke more pronounced innate responses than protein or nucleic-acid vaccines, transient reactogenicity can be misinterpreted as a danger rather than a sign of efficacy. Simultaneously, their capacity to encode personalized neoantigens positions them as leading candidates for individualized cancer vaccines. The field must therefore navigate the paradox of delivering ultra-tailored products to select patients while maintaining broad societal confidence in standardized prophylactic campaigns. Transparent communication, community engagement, and integrated benefit-risk dashboards will be as vital as any genetic modification to sustaining public trust.

Looking ahead, the frontier is likely to shift from single-pathogen targets to combinatorial and even ecosystem-level interventions. Chimeric capsids that evade both human and vector-borne neutralizing antibodies could enable multivalent vaccines protecting against entire viral families. Vectors armed with self-amplifying RNA cassettes may offer dose-sparing solutions that blend the rapid manufacturability of mRNA with the durable immunity typical of live platforms. Finally, coupling vector immunization with real-time pathogen genomics and adaptive trial networks could shorten the interval between threat discovery and population-level immunity from the current twelve months to a matter of weeks.

In summary, viral-vector vaccines have proven their mettle but must evolve on three simultaneous axes—scientific sophistication, manufacturing democratization, and social license to operate—if they are to fulfill their promise as rapid-response tools against future pandemics, stubborn endemic infections, and cancers alike. The field’s next chapter will be written not only in the laboratory but also in factory clean rooms, regulatory meeting rooms, and town hall forums worldwide. With sustained interdisciplinary collaboration and proactive investment, viral-vector platforms are poised to remain at the vanguard of global immunological security for decades to come.

## Figures and Tables

**Table 1:** Comparison of Common Viral Vector Platforms.

Viral Vector	Genome Type	Key Advantages	Key Limitations	Notable Examples/Uses
**Adenovirus (Ad)**	Non-enveloped, dsDNA	- High immunogenicity (strong T- and B-cell responses) - Scalable manufacturing - Thermostability potential	- Pre-existing immunity to common serotypes (e.g., Ad5) can reduce efficacy - Rare risk of VITT (e.g., COVID-19 Ad vaccines)	- Ad5 used in early HIV vaccine trials - ChAdOx1 (Oxford-AstraZeneca COVID-19 vaccine) - Ad26 (Janssen COVID-19 vaccine)
**Adeno-Associated Virus (AAV)**	Non-enveloped, ssDNA	- Low pathogenicity - Long-term episomal persistence - Good safety profile	- Limited cargo capacity (~4.5 kb) - Pre-existing immunity to common AAV serotypes - Typically weaker T-cell responses	- Widely used in gene therapy (e.g., Luxturna) - Under exploration for prophylactic vaccines
**Poxviruses (MVA, NYVAC)**	Enveloped, dsDNA (large genome)	- Historically safe/efficacious (e.g., smallpox); - Large transgene capacity - Strong immunogenicity	- More complex manufacturing - May require specialized BSL facilities - Some reactogenicity in immunocompromised patients	- MVA-based HIV and malaria vaccine candidates - Ervebo’s design concept influenced by poxvirus work
**Vesicular Stomatitis Virus (VSV)**	Enveloped, negative-sense RNA	- Potent humoral and cellular responses - Pseudotyping flexibility (swap surface glycoproteins)	- Concerns about neurovirulence or unintended tissue tropism - Must be sufficiently attenuated for safety	- rVSV-ZEBOV (Ebola vaccine)
**Measles Virus (MV)**	Enveloped, negative-sense RNA	- Established live-attenuated vaccine strain - Strong, long-lasting immunity - Broad population coverage	- Genetic stability and attenuation must be tightly controlled - May need re-engineering in populations with strong anti-measles immunity	- Investigational MV-based vaccines for respiratory pathogens (e.g., COVID-19, RSV)
**Alphavirus Vectors (e.g., Sindbis, Semliki Forest)**	Enveloped, positive-sense RNA	- Self-amplifying RNA replicons allow high antigen expression - Strong innate immune activation	- Potential for high reactogenicity - Attenuation can be challenging - Manufacturing scale-up is still evolving	- Chikungunya vaccine candidates - Self-amplifying RNA (saRNA) platforms
**Lentiviruses (LV)**	Enveloped, ssRNA (retrovirus)	- Sustained antigen expression if integrated - Potent T-cell induction; Integration-defective variants (IDLVs) reduce risks	- Complex manufacturing and higher costs - Biosafety considerations for production (BSL-2/BSL-3) - Potential insertional mutagenesis (if integrative)	- Therapeutic cancer vaccines - Experimental HIV, oncology, and chronic infection vaccines
**Insect-Specific Flaviviruses**	Enveloped, positive-sense RNA	- Replication-deficient in vertebrate hosts - Potential for good safety profile	- Early-stage research - Unknown large-scale manufacturing capacity - May need robust clinical data	- Developing vaccines against mosquito-borne viruses (dengue, Zika, chikungunya)

**Table 2:** maps the major challenges in viral vector vaccine development to a set of practical or proposed mitigation strategies. Challenges vs. Potential Solutions in Viral Vector Vaccine Development.

Challenge	Potential Solutions/Mitigation Strategies
**Pre-Existing Immunity (e.g., to Ad5)**	- Use Alternative/Novel Serotypes: Employ vectors with lower population-level seroprevalence (e.g., Ad26, Ad35, chimp adenoviruses)
	- Pseudotyping or Capsid Engineering: Alter surface proteins to evade neutralizing antibodies
	- Heterologous Prime-Boost: Prime with one vector (e.g., Ad26) and boost with another (e.g., MVA) to avoid anti-vector immunity
**Manufacturing Bottlenecks (Scale-up, QC)**	- Technology Transfer & Licensing: Foster partnerships with regional manufacturers, share standard operating procedures (SOPs) under licensing agreements
	- Single-Use Bioreactors & Platform Processes: Reduce contamination risk, improve flexibility in scaling up
	- Automated Downstream Processing: Chromatography, TFF, and other purification steps that can be standardized for high throughput
**Rare Adverse Events (e.g., VITT in Ad vectors)**	- Improved Purification & Formulation: Minimize free viral DNA or contaminants thought to trigger platelet activation (e.g., PF4 interactions)
	- Enhanced Pharmacovigilance: Rapid detection of safety signals via robust surveillance systems (VAERS, EudraVigilance)
	- Vector Genome Engineering: Remove or modify immunogenic motifs implicated in pathologic clotting cascades
**Equitable Distribution (Global access)**	- Licensing Agreements & IP Waivers: Shorten the timeline to produce vaccines in LMICs
	- Regional Manufacturing Hubs: Decentralize production to reduce dependency on a few facilities worldwide
	- Thermostable Formulations: Lyophilization, novel excipients, or freeze-dried forms reduce cold-chain constraints
**Maintaining Efficacy and Durability**	- Vector Optimization: Incorporate molecular adjuvants, use codon optimization, or add immune-modulatory genes
	- Repeat Boosting Strategies: Heterologous regimens to overcome anti-vector immunity
	- Combination Immunotherapies: Pair vector vaccines with checkpoint inhibitors to sustain T-cell responses (especially in cancer or chronic infections)
**Public Trust & Vaccine Hesitancy**	- Transparent Risk–Benefit Communication: Provide clear data on rare adverse events, emphasize net public health benefits
	- Community Engagement: Partner with local leaders, use culturally appropriate messaging
	- Ongoing Education Campaigns: Increase visibility of scientific evidence via multiple media outlets
